# Comparison of dementia risk between end stage renal disease patients with hemodialysis and peritoneal dialysis - a population based study

**DOI:** 10.1038/srep08224

**Published:** 2015-02-23

**Authors:** Yi-Ting Lin, Ping-Hsun Wu, Mei-Chuan Kuo, Cheng-Sheng Chen, Yi-Wen Chiu, Yi-Hsin Yang, Ming-Yen Lin, Shang-Jyh Hwang, Hung-Chun Chen

**Affiliations:** 1 Department of Family Medicine, Kaohsiung Medical University Hospital, Kaohsiung, Taiwan; 2 Division of Nephrology, Department of Internal Medicine, Kaohsiung Medical University Hospital, Kaohsiung, Taiwan; 3 Department of Psychiatry, Kaohsiung Medical University Hospital, Kaohsiung, Taiwan; 4 Department of Public Health, College of Life Science, Kaohsiung Medical University, Kaohsiung, Taiwan; 5 Graduate Institute of Medicine, Kaohsiung Medical University, Kaohsiung, Taiwan; 6 Faculty of Renal Care, Kaohsiung Medical University, Kaohsiung, Taiwan; 7 Department of Psychiatry, College of Medicine, Kaohsiung Medical University, Kaohsiung, Taiwan; 8 School of Pharmacy, College of Pharmacy, Kaohsiung Medical University, Kaohsiung, Taiwan; 9 Technology Research Center, National Applied Research Laboratories, Taiwan

## Abstract

A higher risk of dementia was reported in patients undergoing maintenance hemodialysis (HD) compared to those undergoing peritoneal dialysis (PD). Selection bias and competing risk of death were not considered in previous studies. The aim of this study was to investigate dementia risk in patients undergoing HD and PD by using the Taiwan Longitudinal Health Insurance Database. We enrolled 52,332 incident HD patients and 3292 incident PD patients who were older than 40 years between January 1, 1998 and December 31, 2007. During the study period, 3775 patients were diagnosed with dementia in the HD group (177.5 per 10,000 person-years incidence rate) and 181 patients in the PD group (145.9 per 10,000 person-years incidence rate). The results revealed that the higher hazard ratio of HD compared with PD for dementia disappeared after controlling for demographic characteristics, propensity score, and competing death risk (subdistribution hazard ratio was 1.086; 95% confidence interval, 0.940–1.255). In conclusion, HD did not increase the risk of dementia in dialysis-dependent patients compared to PD.

Dementia is a disorder of progressive cognitive dysfunction characterized by deterioration in daily life activities and the presence of psychiatric symptoms. It is a major cause of death and disability among elderly individuals in the general population^1^. Recent studies suggest that patients with end-stage renal disease (ESRD) have higher rates of cognitive impairment and dementia than the general population^2^,^3^,^4^,^5^. Dementia further worsens adverse outcomes, including disability, hospitalization, dialysis withdrawal, and mortality^6^,^7^,^8^. ESRD patients share risk factors for dementia with the general population^9^,^10^, and have nephrogenic-related and ESRD treatment-associated risk factors^10^,^11^,^12^. However, unlike the general population, patients with chronic kidney disease and ESRD have vascular dementia equally or more frequently than Alzheimer's disease (AD)^13^,^14^,^15^.

Despite the dementia risk in dialysis patients, the literature is limited regarding the influence of different dialysis modalities, namely hemodialysis (HD) and peritoneal dialysis (PD), on the risk of dementia. The existing literature suggests that the prevalence of cognitive impairment might be lower for patients undergoing PD than HD^16^,^17^,^18^. However, these results may be compromised by small sample sizes, selection bias, cross-sectional designs, and possible confounding factors^12^. Therefore, longitudinal studies are needed to determine whether dialysis modality is an independent risk factor for dementia in ESRD patients. This study's aim was to investigate the risk of dementia in ESRD patients with HD and PD using the National Health Insurance Research Database (NHIRD) in Taiwan. We hypothesized that HD patients would not have an increased risk of dementia compared to PD patients.

## Methods

### Data Source and Study Design

This study is based on the Taiwanese longitudinal health insurance database: the NHIRD. In 1995, Taiwan launched a compulsory social insurance program, the National Health Insurance (NHI), to provide health care for all residents. The database consists of the detailed health care data for over 99% of Taiwan's population.

The NHIRD includes a registry system for “Catastrophic Illnesses,” including ESRD. Insured persons with major diseases can apply for a catastrophic illness registration card from the Bureau of NHI. The database includes all relevant information about the catastrophic illness, including diagnostic codes (in the format of the *International Classification of Disease, Ninth Revision, Clinical Modification* [*ICD-9-CM*]), date of diagnosis, date of death, details of prescriptions, and details of outpatient/inpatient claimed data (for the beneficiaries with catastrophic illnesses). In this study, all cases of dialysis patients were retrieved from the Registry of Catastrophic Illness Database, a subpart of the NHIRD. For each dialysis patient, the information retrieved was validated by two nephrologists through careful examination of the underlying disease, laboratory data (e.g., renal function, nutritional status, and electrolyte levels), renal ultrasonography, and indications for dialysis treatment. For the current retrospective analysis, we included all dialysis patients registered in the Catastrophic Illness Database. In the ethic aspect, the database used consisted of de-identified secondary data, the study met the requirements of the “Personal Information Protection Act” in Taiwan. The data were analyzed anonymously and the need for informed consent was waived approved by institution of review board.

### Study Cohorts

We selected patients diagnosed with ESRD and who received HD and PD for more than 90 days between January 1, 1998, and December 31, 2007. A diagnosis of ESRD was defined as having catastrophic illness registration cards for ESRD (*ICD-9-CM* code 585). We excluded individuals who were younger than 40 years (n = 4,385), had received renal transplantation either before or after dialysis (n = 479), and were diagnosed with dementia before dialysis (n = 3,494). The renal transplant rate is low in Taiwan; therefore, we excluded all patients who received transplants to avoid any confounding effects^19^,^20^,^21^. The modality that was used regularly after 90 days of dialysis was considered the initial treatment modality; patients who changed dialysis modality were classified by their initial treatment modality.

### Comorbidities and Exposure to Confounding Medications for Propensity Score Estimation

We retrieved background information on patients' comorbidities one year before their first dialysis from the NHI database (Supplemental Table S1). Participants' use of other medications during the observation period is listed in Supplemental Table S2. Medication exposure was defined as an average frequency of at least one tablet per month. Comorbidities, medications, age, and sex were applied in the logistic regression models as propensity scores. Propensity score analyses were used to statistically control for the differences between the HD and PD groups on baseline patient characteristics.

### Main Outcome Measurements and Follow-up

The main outcome variable was dementia (*ICD-9-CM* code: 290, 291.2, 294.1, 331.0, 331.1, and 331.2). Dementia classification required at least two records of outpatient visits or one hospital admission, and a diagnosis made by a neurologist or psychiatrist. We chose to analyze all types of dementia for a number of reasons. Indeed, the differentiation between AD and vascular dementia is not clear in certain situations^22^, and vascular dementia is considered a heterogeneous group of syndromes^23^. There is also considerable overlap in risk factors, clinical features, and radiographic and neuropathological findings between AD and vascular dementia. Moreover, the relationship between vascular lesions may modify the course of AD, and this relation is bidirectional^24^. We only performed “intent-to-treat” survival analysis and spared “as–treated” when comparing the two modalities. We followed this procedure because it was difficult to define the rule to assign the event to previous or latter modality once dementia was diagnosed after the modality shift and the rate of modalities transferred was a small portion of the long-term dialysis patients in Taiwan^20^. In the present study, there were only 1814 patients (3.26%) who changed dialysis modality during the observation period.

The follow-up period started on the index date of incident dialysis and continued for dementia diagnosis until the date of death or until December 31, 2008. A death event was identified if patients' discharge status was coded as “death,” dates of death were claimed by catastrophic illness registration files, or patients unenrolled from the NHI program and who had not enrolled in the NHI beneficiary registry files. As the NHI is a compulsory plan, it would be rare that a patient would unenrol unless death had occurred.

### Statistical Analysis

The continuous data were analyzed via Student's *t*-test; the categorical data were analyzed via chi-squared tests. To evaluate the performance of propensity score adjustment in controlling for confounding, logistic regressions with dementia as outcome were performed for each of the baseline characteristics, comorbidities, and medication use for propensity score^25^. We then calculated the crude and subdistribution hazard ratios (HRs) controlling for propensity score strata in deciles and their 95% CIs comparing HD versus PD initiators using proportional hazards regression.

The occurrence of dementia in the two different dialysis modality groups has a competing risk for death; therefore, we used the cumulative incidence competing risk (CICR) method^26^ to estimate the cumulative incidence rates of dementia in the two groups. We also tested for the fulfillment of the assumption of proportional hazards by creating interactions between predictors and survival time (the *P* value for proportionality is 0.140). We then applied the proportional hazards model for the subdistribution of a competing risk to estimate the subdistribution hazard ratios and 95% confidence intervals (CIs) in relation to the primary outcomes^27^. The relative dementia risk of the two dialysis modalities was further compared across different strata (Tables 1 & 2). Analyses were performed using the SAS statistical package (version 9.3; SAS Institute Inc, www.sas.com). Calculations of cumulative incidence in the competing risk analysis were undertaken using the “cmprsk” package R (http://cran.r-project.org/web/packages/cmprsk/index.html). All statistical tests were two-tailed. A *P*-value < 0.05 was considered statistically significant.

## Results

### Baseline Characteristics of HD groups and PD groups

**Figure 1:**
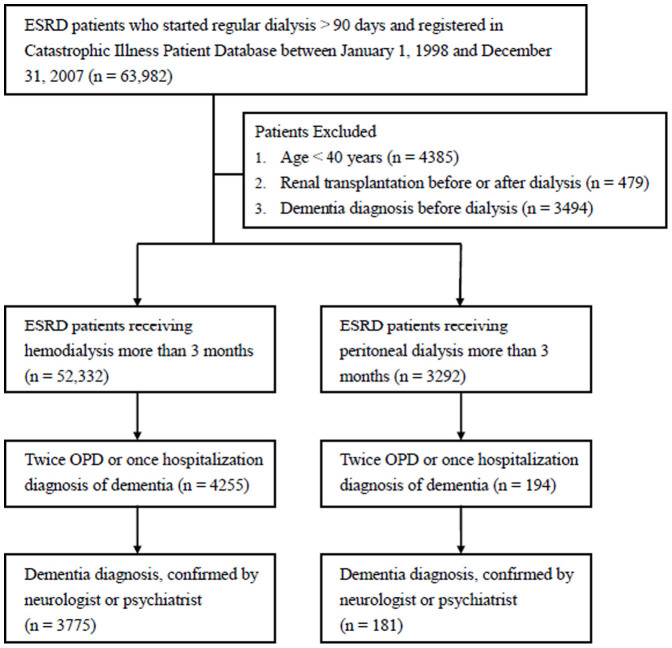
Schematic diagram of study participant selection and dementia identification in both HD and PD group.

Figure 1 presents a flow chart of the study. There were 63,982 incident patients undergoing dialysis for more than 3 months between 1998 and 2007. After removing patients based on the aforementioned exclusion criteria, 55,264 eligible patients were enrolled. Of these eligible patients, 52,332 received HD and 3,292 received PD for more than 3 months. Table 1 compares the clinical characteristics of the HD group and the PD group before the propensity score analyses. The patients in the HD group were older; more likely to be male; living in urban areas; of a lower socioeconomic status; more likely to have diabetes mellitus, heart failure, peripheral artery disease, chronic obstructive pulmonary disease, osteoarthritis, chronic liver disease, peptic ulcer disease, malignancy, or alcohol dependence; and less likely to have a history of other underlying diseases, including hypertension, hyperlipidemia, systemic lupus erythematosus, and gout than the PD group. In addition, a significantly higher proportion of the patients in the PD group received dipyridamole, angiotensin receptor blockers, beta-blockers, calcium-channel blockers, statins, fibrates, benzodiazepines, hypnotics, anti-epileptics, and uric acid lowering agents. However, the patients in the HD group received more oral antidiabetic agents, traditional nonsteroidal anti-inflammatory drugs, histamine-2 receptor antagonists, and antipsychotic agents than the PD group. There was no significant difference in these characteristics between the two dialysis modalities groups after the propensity score adjustment.

### Cumulative Incidence and Hazard ratio of Dementia between HD and PD during Follow-up

**Figure 2:**
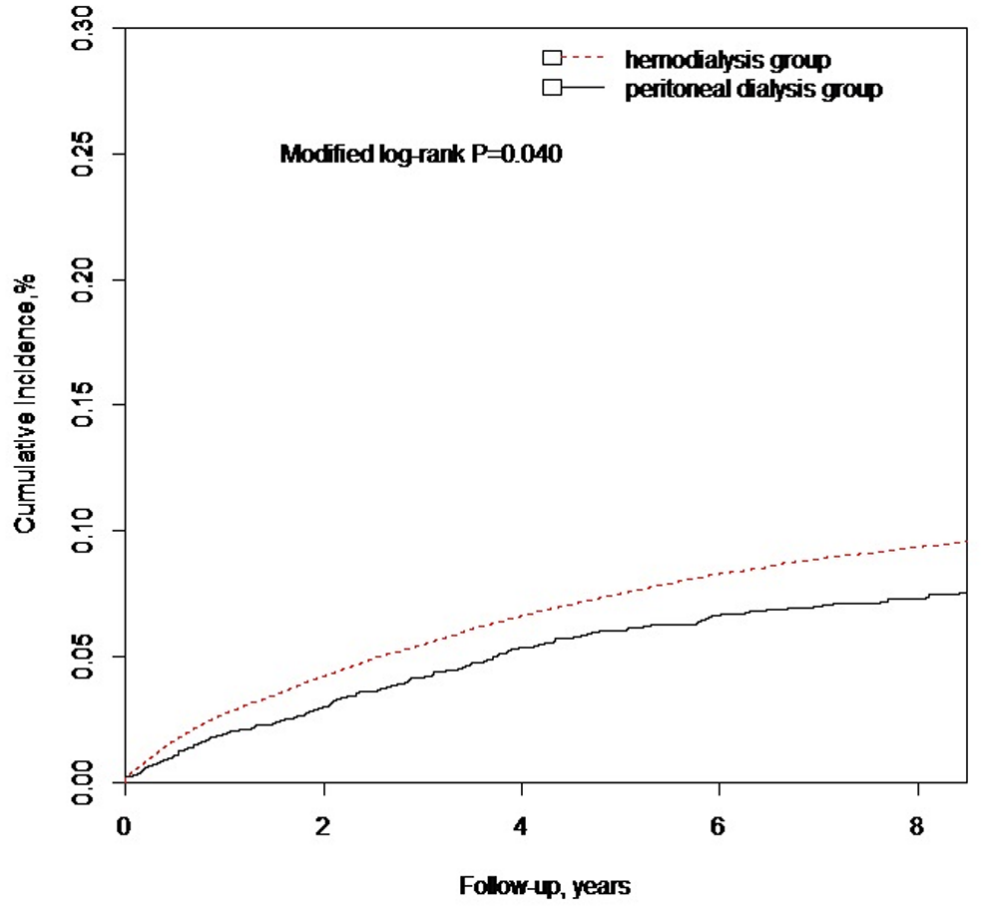
Cumulative incidences of dementia between hemodialysis and peritoneal dialysis. For the cumulative incidences of dementia, cumulative incidence competing risk method (Gray methods) were performed by conducting calculations and comparisons using competing risk data.

A total of 3775 dementia case in the HD group and 181 cases in the PD group had been identified in the observation period. Table 3 presents information on follow-up duration, dementia events, and dementia rate. The mean follow up years were 4.07 ± 3.10 years in the HD group and 3.79 ± 3.06 years in the PD group. The median follow up years were 3 years in the HD group and 3 years in the PD group. Compared with HD, PD was associated with similar survival rates in the present study (data not shown). The crude incidence rate of dementia per 1,000 person-years was 17.75 (95% CI, 17.19–18.33) for the HD group initiators and 14.59 (95% CI, 12.54–16.88) for the PD group (Table 3). Using CICR method with considering for competing risk of mortality, the cumulative incidence rates for dementia occurrence were significantly higher in the HD group (7.21%) than in the PD group (5.50%) (modified Log-rank *P* = 0.04; Figure 2). Table 4 presents the univariate and multivariable Fine and Gray regression model analyses. The unadjusted HR for the occurrence of dementia in the HD group compared with the PD group (reference group) was 1.24 (95% CI, 1.07–1.44; *P* < 0.001). Death before the occurrence of dementia was defined as competing mortality. A multivariable analysis using the proportional hazards model for the subdistribution of a competing risk was further used for competing mortality. In this model (Model 1), the HD group showed a higher risk of dementia (subdistribution HR [sHR], 1.30, 95% CI, 1.13–1.50; *P* < 0.001) than the PD group. Compared with the PD group, the dementia risk with the HD group was not significant after additionally adjusting for age, sex, urbanization level, and socioeconomic status (sHR, 1.06, 95% CI, 0.92–1.23; *P* = 0.425; Model 2). The dementia risk was still not statistically significant difference between the HD group and the PD group after controlling for baseline propensity scores and competing death risk (sHR, 1.09, 95% CI, 0.94–1.26; *P* = 0.264; Model 3).

### Subgroup Analyses

**Figure 3:**
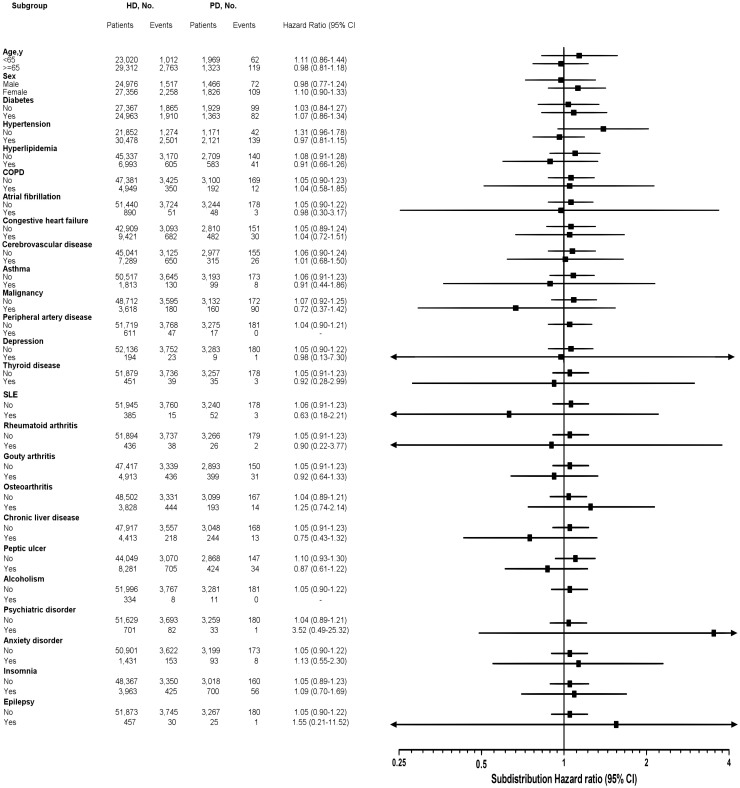
Stratified analysis for dementia between hemodialysis and peritoneal dialysis. The risk of dementia between hemodialysis and peritoneal dialysis (presented by subdistribution hazard ratios and 95% confidence intervals in subgroups of patients after adjusting for propensity score and using the competing risk approach) is shown, stratified by the baseline characteristics.

Figure 3 shows the results of the multivariate stratified analysis. In each stratum, the sHR was compared between the HD group and the PD group. The HD group was not associated with an increased risk of dementia in any of the subgroups. The stratified analysis sHRs were used a competing death risk approach and adjusted for baseline propensity scores. These observations further confirmed the same not statistically significant different dementia risk between the HD group and the PD group in incident dialysis patients.

## Discussion

Our study compared the risk of dementia between HD and PD groups while accounting for the competing risk of death and other covariates as propensity scores (Tables 1 and 2). The incidence rate of dementia, crude HR, and competing death risk subdistribution HR were higher in the HD group than the PD group. However, the results indicated that the higher hazard ratio of HD, compared with PD, for dementia disappeared after adjusting for demographic characteristics, including age, sex, urbanization level, and socioeconomic status. To our knowledge, this is the first study to demonstrate that there is no difference in risk of dementia occurrence after dialysis between HD and PD from a nationwide cohort study.

We also found an equal hazard risk between HD and PD for developing dementia. Importantly, this was inconsistent with previous work indicating a higher risk for HD^16^,^17^,^18^. However, previous studies consisted of investigations with small sample sizes, cross-sectional designs, and/or unadjusted comorbidities and demographic characteristics. These methodologies may be flawed given that cross-sectional observation studies account for both the effect before and after dialysis. Furthermore, each dialysis modality has a different effect on patients' physical, psychological, and social health. Thus selection bias may exist if researchers do not adjust for confounding factors. The longitudinal design used herein may better reflect the dialysis procedure and adjust for confounding factors.

Our results confirm a different demographic distribution between patients choosing HD and PD (Table 1). The HD patients tended to be elderly, of lower socioeconomic status, and living in urban areas; the first two demographic features are risk factors for dementia^6^,^28^. Therefore, we suggest that HD was mistakenly considered a risk factor for dementia over PD because previous studies did not account for confounding demographic factors.

The neuropathology of cognitive impairment and dementia in ESRD is not well-known. Cerebrovascular disease may play an important role. Indeed, the brain and kidneys have many common anatomic and vasoregulatory features, being low resistance end organs exposed to a high volume of blood flow and susceptible to vascular damage^29^. Hence, dementia and ESRD are reflections of vascular injury in different end organs and share a common pathogenesis^10^. However, ischemic stroke may be why vascular dementia exceeds the portion of AD among dialysis patients. However, when HD and PD patients are compared, there is no significant difference in developing ischemic stroke^30^,^31^; this may explain why there was no significantly different risk for dementia in HD and PD patients in this study.

Additionally, other potential mechanisms, including direct neuronal injury by uremic toxins, could also be involved in dementia risk among ESRD patients. In general, dialysis efficacy, such as uremic toxin removal, is different between HD and PD. However, the persistence of cognitive impairment despite clinically adequate dialysis dose delivery indicates that other factors also contribute to brain dysfunction. Furthermore, frequent HD did not improve cognition in a randomized clinical trial^32^,^33^ and did not suggest that uremic toxins are the major cause of cognitive impairment. Thus, cerebrovascular disease remains a powerful risk factor for the development of cognitive impairment, especially in dialysis patients.

Although the risk for dementia is similar in both modalities, some mechanisms between them may differ. Beyond the cerebrovascular events related to dementia risk, the HD process results in rapid changes in blood pressure, rapid and large intravascular volume loss, fluid shifts leading to subclinical cerebral edema, decreased cerebral perfusion, and cerebral ischemia; each may contribute to cognitive impairment^34^,^35^. Thus, the massive, uncontrollable hemodynamic and metabolic changes in the brain during HD sessions may contribute to cognitive decline in HD patients. PD does not involve the same rapid fluid and electrolyte shifts; however, fluid overloading and secondary metabolic disorders from glucose-based dialysate used in PD may contribute to cognitive impairment.

In addition, white matter disease (leukoaraiosis) is present in many PD^36^ and HD patients^36^; this is related to vascular degenerative morphology consistent with chronic hypoxia and vascular hypoperfusion. Hence, white matter disease is associated with an increased risk of stroke, disability, and cognitive impairment and decline^37^,^38^. Thus, it is possible that there is a similar risk, but via a different pathway for PD and HD patients. Therefore, the prevention of cerebrovascular disease may be a crucial for the prevention of dementia in both PD and HD patients.

The present study had a number of strengths, including a nationally representative, non-selective cohort of dialysis patients from a well-established research database. The assessment of the HD groups and PD groups was accurate because NHI is a compulsory and universal healthcare system with a high coverage rate in Taiwan. Indeed, patient dropout was avoided and recall bias minimized because all claims from different medical institutes from 1998–2007 were obtained for analysis. Furthermore, the diagnosis of dementia was strictly defined by neurologists or psychiatrists after the maintenance dialysis. Thus, diagnosis accuracy was comprehensive.

Nevertheless, there were limitations that warrant consideration. The study was retrospective and based on a review of the medical records, not on formal cognitive functioning tests. Diagnosis based on ICD-9 coding is likely to underestimate the true number of dementia cases in our study because dialysis patients are rarely assessed for dementia. Thus, there was higher portion of not confirmed dementia cases in HD than PD group according to the way we used to identify dementia (11.3% in HD group vs. 6.8% in PD group as shown in Figure 1). Although this could be a random result since HD and PD had similar comorbidities and care facilities, bias of underestimate the risk for dementia could exist. In order to define the diagnosis of dementia more precisely, the one who had ICD-9 coding given by neurologist or psychiatrist was identified as dementia patient. Neurologist or psychiatrist was well trained and the most qualified to evaluate the diagnosis of dementia in our country. The diagnosed dementia rate by neurologist or psychiatrist was 7.21% in the HD group and 5.50% in the PD group; this is similar to dementia diagnoses in the Dialysis Outcomes and Practice Patterns Study^6^. Additionally, the incidence of dementia in this study was higher than chronic kidney disease^39^ and the general population^40^ (17.75 per 1,000 person-years for the HD group, 14.59 per 1,000 person-years for the PD group). Dementia coding has reportedly low sensitivity but high specificity^41^,^42^, and physician recognition and documentation of dementia underestimates the true prevalence of dementia^43^. Therefore, it is likely that patients identified as having dementia in our study did have dementia, but also that there were patients with dementia who were not identified. Undifferentiated misclassification generally biases results towards null findings.

Additionally, the diagnosis of the various comorbidities was based on claims data and *ICD-9-CM* codes potentially associated with a misclassification bias. However, the NHI Bureau of Taiwan performs regular audits on the quality of data captured and imposes heavy penalties for outlier charges or malpractice. Moreover, the NHIRD lacks information about other potential confounding factors–such as smoking, physical inactivity, genetic factors, family history, laboratory data, dialysis dose, and hemodynamic stability during dialysis sessions–which is a limitation. Furthermore, information regarding the degree or stage of dementia was unavailable, and dementia subtypes were not considered in the analyses herein. Finally, this study included Taiwanese patients, meaning that the results may not be generalizable to other populations because of the differences in dementia incidence, subtypes, and pathogenesis across countries.

## Conclusion

This large nationwide population-based cohort study suggests no significant difference in dementia risk among Taiwanese HD patents and PD patients.

## Author Contributions

Conceived and designed the experiments: Y.T.L., P.H.W., M.C.K., C.S.C. and Y.W.C. Performed the experiments: Y.T.L. and P.H.W. Analyzed the data: Y.T.L., Y.H.Y. and M.Y.L. Prepare Tables and Figure: Y.T.L. and P.H.W. Wrote the paper: Y.T.L., P.H.W., C.S.C., Y.W.C., S.J.H. and H.C.C. All authors reviewed the manuscript.

## Supplementary Material

Supplementary Information Supplementary table

## Figures and Tables

**Table 1:** Baseline patient characteristics and comorbidities between hemodialysis and peritoneal dialysis

	Hemodialysis (n = 52,332)		Peritoneal dialysis (n = 3292)		
Characteristic	N	%	N	%	*P* value
**Age at cohort entry**					<0.001
40–54	10,346	19.8	1101	33.4	
55–64	12,674	24.2	868	26.4	
65–74	16,390	31.3	777	23.6	
> = 75	12,922	24.7	546	16.6	
**Sex**					<0.001
Male	24,976	47.7	1466	44.5	
Female	27,356	52.3	1826	55.5	
**Initiation year**					<0.001
1997–1999	12,545	24	703	21.4	
2000–2002	14,177	27.1	768	23.3	
2003–2005	15,784	30.2	980	29.8	
2006–2008	9826	18.8	841	25.5	
**Urbanization level**					<0.001
City area	14,390	27.5	709	21.5	
Rural area	37,942	72.5	2583	78.5	
**Socioeconomic status**					<0.001
Low economics	22,084	42.2	1331	40.4	
Moderate economics	14,714	28.1	797	24.2	
High economics	15,534	29.7	1164	35.4	
**Comorbidities**					
Diabetes mellitus	24,963	47.7	1363	41.4	<0.001
Hypertension	30,478	58.2	2121	64.4	<0.001
Hyperlipidemia	6993	13.4	583	17.7	<0.001
Ischemic heart disease	10,745	20.5	679	20.6	0.899
Heart failure	9421	18	482	14.6	<0.001
Atrial fibrillation	890	1.7	48	1.5	0.294
Peripheral artery disease	611	1.2	17	0.5	<0.001
Cerebrovascular disease	7289	13.9	315	9.6	<0.001
COPD	4949	9.5	192	5.8	<0.001
Asthma	1813	3.5	99	3	0.163
Thyroid disease	451	0.9	35	1.1	0.229
SLE	385	0.7	52	1.6	<0.001
Rheumatoid arthritis	436	0.8	26	0.8	0.790
Osteoarthritis	3828	7.3	193	5.9	0.002
Gout	4913	9.4	399	12.1	<0.001
Chronic liver disease	4413	8.4	244	7.4	0.040
Peptic ulcer disease	8281	15.8	424	12.9	<0.001
Malignancy	3618	6.9	160	4.9	<0.001
Alcohol dependence	334	0.6	11	0.3	0.031
Psychotic disorder	701	1.3	33	1	0.100
Depressive disorder	194	0.4	9	0.3	0.369
Anxiety disorder	1431	2.7	93	2.8	0.758
Sleep disorder	3963	7.6	274	8.3	0.116
Seizure disorder	457	0.9	25	0.8	0.494

Abbreviations: COPD, chronic obstructive pulmonary disease; SLE, systemic lupus erythematosus.

**Table 2:** Baseline patient medications prescription between hemodialysis and peritoneal dialysis

	Hemodialysis (n = 52,332)		Peritoneal dialysis (n = 3292)		
Medications use	N	%	N	%	*P* value
Antiplatelets	11,986	22.9	740	22.5	0.573
Anticoagulants	703	1.3	40	1.2	0.534
Dipyridamole	12,935	24.7	906	27.5	<0.001
Nitrates	10,804	20.6	687	20.9	0.759
ACEIs	12,375	23.6	780	23.7	0.951
ARBs	10,128	19.4	841	25.5	<0.001
Beta-blockers	16,174	30.9	1346	40.9	<0.001
Thiazides	10,736	20.5	675	20.5	0.988
CCBs	26,391	50.4	1928	58.6	<0.001
Statins	7915	15.1	734	22.3	<0.001
Fibrates	3226	6.2	271	8.2	<0.001
Oral antidiabetic agents	15,213	29.1	868	26.4	<0.001
Insulin	7543	14.4	502	15.2	0.186
Traditional NSAIDs	9331	17.8	480	14.6	<0.001
COX-2 inhibitors	2525	4.8	145	4.4	0.274
PPIs	3245	6.2	208	6.3	0.786
H-2 receptor antagonists	2651	5.1	136	4.1	0.017
Antipsychotic agents	1236	2.4	57	1.7	0.020
Antidepressants	3200	6.1	216	6.6	0.301
Benzodiazepines	8114	15.5	575	17.5	0.003
Hypnotics	7479	14.3	558	17	<0.001
Antiepileptics	4113	7.9	330	10	<0.001
Uric acid lowering agents	6885	13.2	600	18.2	<0.001

Abbreviations: ACEIs, angiotensin-converting-enzyme inhibitors; ARBs, angiotensin receptor blockers; CCBs, calcium-channel blockers; traditional NSAIDs, traditional nonsteroidal anti-inflammatory drugs; COX-2 inhibitors, cyclooxygenase-2- selective inhibitors; PPIs, proton-pump inhibitors; H2- receptor antagonists, histamine-2 receptor antagonists.

**Table 3:** Follow-up duration, dementia events, and crude incidence rate of dementia among hemodialysis groups and peritoneal dialysis groups

Clinical outcome	Hemodialysis (n = 52,332)	Peritoneal dialysis (n = 3292)
Total follow-up person-years	212,641	12,405
Numbers of dementia cases	3775	181
Mean follow-up time ± SD (years)	4.07 ± 3.10	3.79 ± 3.06
Median follow-up time (IQR) (years)	3.00 (1.00–7.00)	3.00 (1.00–7.00)
Incidence rate per 1,000 person-years (95% CI)	17.75 (17.19–18.33)	14.59 (12.54–16.88)

Footnote: CI, confidence interval.

**Table 4:** Hazard ratio for dementia comparing hemodialysis and peritoneal dialysis in various analytical models

	Outcome of dementia comparing hemodialysis and peritoneal dialysis (peritoneal dialysis as reference group)	
Various analytical models	Crude HR (95% CI)	Subdistribution HR^a^ (95% CI)
Model 1	1.241 (1.069–1.440)	1.302 (1.128–1.504)
Model 2	—	1.060 (0.918–1.225)
Model 3	—	1.086 (0.940–1.255)

^a^use the competing risk approach.

Model 1: use the competing risk approach.

Model 2: adjustment for age, sex, urbanization level, socioeconomic status, and use the competing risk approach.

Model 3: adjustment for baseline propensity score and use the competing risk approach.
