# Supplementary material for: Comparison of dementia risk between end stage renal disease patients with hemodialysis and peritoneal dialysis - a population based study
Source: Sci Rep. 2015 Feb 23;5:8224. doi: 10.1038/srep08224 (PMC4340159; doi:10.1038/srep08224)
Supplement: Supplementary Information — Supplementary table [file srep08224-s1.pdf]

Manuscript Title: Comparison of dementia risk between end stage renal disease patients with hemodialysis and peritoneal dialysis - a population based study

Authors, Institution and Affiliations:

Yi-Ting Lin[1,4], Ping-Hsun Wu[2,5], Mei-Chuan Kuo[2,6], Cheng-Sheng Chen[3,7], Yi-Wen Chiu[2,6], Yi-Hsin Yang[8], Ming-Yen Lin[2,9], Shang-Jyh Hwang[2,6], Hung-Chun Chen[2,6]

[1]Department of Family Medicine, [2]Division of Nephrology, Department of Internal Medicine, [3]Department of Psychiatry, Kaohsiung Medical University Hospital, Kaohsiung, Taiwan;

[4]Department of Public Health , College of Life Science [5]Graduate Institute of Medicine, [6]Faculty of Renal Care, [7]Department of Psychiatry, College of Medicine, [8]School of Pharmacy, College of Pharmacy, Kaohsiung Medical University, Kaohsiung, Taiwan

[9]Technology Research Center, National Applied Research Laboratories, Taiwan

Corresponding author:

Yi-Wen Chiu, M.D

Division of Nephrology, Department of Internal Medicine, Kaohsiung Medical University Hospital

No.100, Tzyou 1st Road Kaohsiung 807, Taiwan

Telephone number: 886-7-3121101 ext 7351

Fax number: 886-7-3228721

E-mail address: chiuyiwen@kmu.edu.tw

Supplementary Table S1. ICD-9-CM codes used to identify clinical conditions

Diagnosis	Corresponding ICD-9-CM codes
Hemodialysis	【585】plus procedure code 58001C, 58019C, 58020C, 58021C, 58022C, 58023C, 58024C, 58025C, 58026C, 58027C, 58029C last for more than 3 months
Peritoneal dialysis	【585】plus PD solution using for more than 3 months
Dementia	【290】【291.2】【294.1】【331.0】【331.1】【331.2】
Diabetes mellitus	【250】
Hypertension	【401】~【405】
Hyperlipidemia	【272】
Coronary artery disease	【410】~【414】
Heart failure	【398.91】【402.01】【402.11】【402.91】【404.01】【404.03】【404.11】【404.13】【404.91】【404.93】【428】
Atrial fibrillation	【427.3】
Peripheral artery disease	【440.2】~【440.4】【443.9】
Cerebrovascular disease	【430】~【438】
Chronic obstructive pulmonary disease	【491】【492】【496】
Asthma	【493】
Thyroid disease	【242】【244】【245】
Systemic lupus erythematous	【710】

Rheumatoid arthritis	【714】
Osteoarthritis	【715】
Gout	【274】
Chronic liver disease	【571】【572.2】~【572.8】【573.1】~【573.3】【456.0】~【456.2】
Peptic ulcer disease	【531】 ~ 【534】
Malignancy	【140】 ~ 【208】
Alcoholism	【291】【303】【305.0】【357.5】【425.5】【571.0】【571.1】 【571.2】 【571.3】 【980.0】 【V11.3】
Psychotic disorder	【290.8】【290.9】【295】【297】~【299】【780.1】
Depressive disorder	【296.2】 【296.3】 【296.82】 【300.4】 【309.0】【309.1】 【311】
Anxiety disorder	【300.0】 ~ 【300.3】
Sleep disorder	【307.4】 【780.5】
Seizure disorder	【345】 【780.3】

Footnotes: ICD, international classification of disease

Supplementary Table S2. Drugs (Anatomical Therapeutic Chemical code) concomitant prescriptions in the present study

Drug type	ATC classification system codes	Drug name
Antiplatelets	B01AC04, B01AC05, B01AC06	Clopidogrel, Ticlopidine, Acetylsalicylic acid (Aspirin)
Anticoagulants	B01AA03	Warfarin
Dipyridamole	B01AC07	Dipyridamole
Nitrates	C01DA02, C01DA04, C01DA14, C01DA52	Glyceryl trinitrate, Isosorbide dinitrate, Isosorbide mononitrate
Angiotensin-converting enzyme inhibitors (ACEIs)	C09A, C09B	Captopril, Enalapril, Lisinopril, Perindopril, Ramipril, Quinapril, Benazepril, Cilazapril, Fosinopril, Imidapril
Angiotensin receptor blockers (ARBs)	C09C, C09D	Candesartan, Irbesartan, Losartan, Olmesartan, Telmisartan, Valsartan
Beta-blockers	C07A	Labetalol, Pindolol, Acebutolol, Alprenolol, Atenolol, Betaxolol, Bisoprolol, Carteolol, Carvedilol, Nadolol, Metoprolol, Oxprenolol, Propranolol, Sotalol, Timolol, Metipranolol, Esmolol
Thiazides	C03AA03, C03AA06	Hydrochlorothiazide, Trichlormethiazide
Calcium channel blockers (CCBs)	C08C, C08D, C08E	Nifedipine, Nicardipine, Felodipine, Amlopidine, Isradipine, Diltiazem, Verapamil
Statins	C10AA	Atorvastatin, Fluvastatin, Lovastatin, Pravastatin, Rosuvastatin, Simvastatin
Fibrates	C10AB	Bezafibrate, Clofibrate, Etofibrate, Gemfibrozil, Fenofibrate, Simfibrate
Oral antidiabetic agents	A10B	Acarbose, Acetohexamide, Buformin, Chlorpropamide, Gliclazide, Glimepiride, Glipizide, Gliquidone, Glyburide, Metformin, Nateglinide, Pioglitazone, Repaglinide, Rosiglitazone, Tolazamide, Tolbutamide,

		Sitagliptin, Miglitol
Insulin	A10A	Insulin human, Insulin zinc crystal, Insulin chromatograp, Insulin monocomponem, Insulin isophane, Insulin protamine, Insulin lispro, Insulin glargine, Insulin aspart, Insulin glulisine, Insulin detemir
Traditional nonsteroidal anti-inflammatory drugs (Traditional NSAIDs)	M01AA, M01AB, M01AC, M01AE, M01AG, M01AX	Acemetacin, Aloclofenac, Alminoprofen, Benzydamine, Diclofenac, Diflunisal, Etodolac, Etofenamate, Fenbufen, Flufenamate, Flufenamic acid, Flurbiprofen, Ibuprofen, Iclofenac, Indomethacin, Ketoprofen, Ketorolac, Meclofenamate, Meclofenamic acid, Naproxen, Piroxicam, Spirin, Sulindac, Niflumic acid, Tenoxicam, Tiaprofenic acid, Tiaramide, Tolmetin, Tolfenamic acid, Mepirizole
Cyclooxygenase-2- selective inhibitors (COX-2 inhibitors)	M01AH	Celecoxib, Meloxicam, Rofecoxib, Etoricoxib, Nimesulide
Proton pump inhibitors (PPIs)	A02BC	Omeprazole, Esomeprazole, Pantoprazole, Lansoprazole, Rabeprazole
H-2 receptor antagonists	A02BA	Cimetidine, Ranitidine, Famotidine
Antipsychotic agents	N05A	chlorpromazine, clopenthixol, clothiapine, flupenthixol, fluphenazine, haloperidol, levomepromazine, loxapine, methotrimeprazine, perphenazine, pimozide, pipotiazine, prochlorperazine, sulpiride, trifluoperazine, thioridazine, amisulpride, aripiprazole, clozapine, olanzapine, quetiapine, risperidone, ziprasidone, and zotepine
Antidepressants	N06A	Fluoxetine, Citalopram, Paroxetine, Sertraline, Fluvoxamine,Escitalopram, Imipramine, Clomipramine, Amitriptyline, Doxepin, Dosulepin, Maprotiline, Melitracen, Nortriptyline, Moclobemide, Duloxetine, Milnacipran, Venlafaxine, Trazodone, Mirtazapine, Bupropion

Benzodiazepines	N05B	Diazepam, Chlordiazepoxide, Medazepam, Oxazepam, Clorazepate, Lorazepam, Bromazepam, Clobazam, Alprazolam, Nordazepam, Fludiazepam
Hypnotics	N05C	Secobarbital, Flurazepam, Nitrazepam, Flunitrazepam, Estazolam, Triazolam, Midazolam, Brotizolam, Zopiclone, Zolpidem, Zaleplon, Dexmedetomidine
Antiepileptics	N03A	Phenobarbital, Phenytoin, Clonazepam, Carbamazepine, Oxcarbazepine, Valproic acid, Vigabatrin, Tiagabine, Lamotrigine, Topiramate, Gabapentin, Levetiracetam, Pregabalin
Uric acid lowering agents	M04AA, M04AB	Allopurinol, Febuxostat, Probenecid, Sulfinpyrazone, Benzbromarone
